# The role of epigenetics in hypothalamic energy balance control: implications for obesity

**DOI:** 10.15698/cst2019.07.191

**Published:** 2019-06-05

**Authors:** Arnaud Obri, Marc Claret

**Affiliations:** 1 Neuronal Control of Metabolism Laboratory, Institut d'Investigacions Biomèdiques August Pi i Sunyer (IDIBAPS), 08036 Barcelona, Spain.; 2 Centro de Investigación Biomédica en Red (CIBER) de Diabetes y Enfermedades Metabólicas Asociadas (CIBERDEM), 08036 Barcelona, Spain.

**Keywords:** obesity, hypothalamus, epigenetic, energy balance, AgRP neurons, POMC neurons

## Abstract

Despite enormous social and scientific efforts, obesity rates continue to increase worldwide. While genetic factors contribute to obesity development, genetics alone cannot explain the current epidemic. Obesity is essentially the consequence of complex genetic-environmental interactions. Evidence suggests that contemporary lifestyles trigger epigenetic changes, which can dysregulate energy balance and thus contribute to obesity. The hypothalamus plays a pivotal role in the regulation of body weight, through a sophisticated network of neuronal systems. Alterations in the activity of these neuronal pathways have been implicated in the pathophysiology of obesity. Here, we review the current knowledge on the central control of energy balance with a focus on recent studies linking epigenetic mechanisms in the hypothalamus to the development of obesity and metabolic disorders.

## INTRODUCTION

The prevalence of obesity continues to increase worldwide. This trend is of concern because of its dramatic economic impact, concomitant decreased lifespan and increased comorbidities, such as hypertension, cardiovascular diseases, type 2 diabetes (T2D) and cancer [1–3]. Only in Europe alone, the (direct and indirect) cost of obesity is estimated to be around 81 billion of euros per year.

The most basic definition of obesity refers to an excessive and/or abnormal accumulation of fat. Obesity is considered to be the consequence of an imbalance between energy intake and expenditure [6]. Thus, altered feeding behavior (chronic overeating) and a sedentary lifestyle (chronic low energy expenditure) are important contributors to the development of overweight and obesity.

The brain, and in particular the hypothalamus, plays an essential role in maintaining energy homeostasis. Specific neuronal circuits in the hypothalamus sense and decode multiple nutritional, hormonal and metabolic cues to fine-tune food intake and energy expenditure. However, environmental factors, such as the diet, physical activity or exposure to certain chemicals, can impair the hypothalamic mechanisms controlling appetite and energy balance [7–11]. At the molecular level, epigenetic processes might play a fundamental role in the complex interactions between environment and energy imbalance seen in obesity [12].

In this review, we discuss epigenetic determinants in hypothalamic pathways controlling energy homeostasis and its association with the development of obesity and metabolic syndrome.

## OBESITY IS AT THE INTERPLAY BETWEEN GENETIC AND ENVIRONMENTAL FACTORS

**Figure 1:**
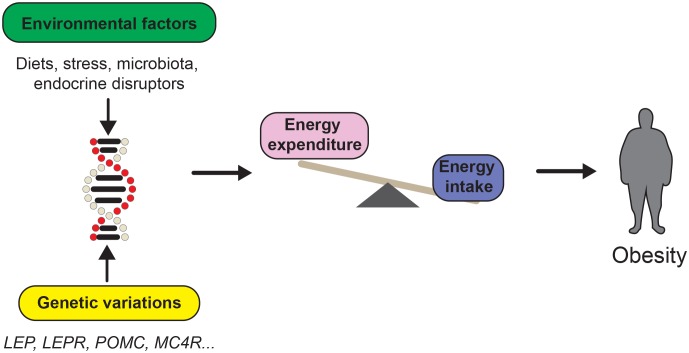
FIGURE 1: Obesity is at the interplay between genetic and environmental factors. The development of obesity is influenced by genetic and environmental factors. The study of monogenic obesity has led the discovery of several obesity susceptibility genes such as *Proopimelanocortin* (POMC), *Melanocortin receptor 4* (*MC4R*) or *Leptin* (*LEP*) among others. However, diverse environmental factors such as the diet, combined with genetic variations, also influence energy balance control.

Energy imbalance is an important contributor of body weight gain; however, the pathophysiology of obesity has proven to be much more complex. Research into monogenic obesity has resulted in the identification of single genes that dramatically affect body weight through hypothalamic pathways [14–17]. Yet, mutations in those genes are relatively rare and account for only ≈5% of obese patients [18]. Genome-wide association studies (GWAS) have emerged as a valuable tool to identify novel genetic factors contributing to obesity [19, 20]. GWAS have identified numerous single nucleotide polymorphisms (SNPs) associated with body mass index (BMI), and again have highlighted the importance of neuronal pathways to obesity [19, 21]. However, genetics alone cannot explain the rather recent and steadfast increase in worldwide obesity rates. At this point, it is clear that obesity stems from the interaction of susceptibility genes with multiple environmental factors (**Figure 1**).

There are critical periods in the prenatal and perinatal phases that are particularly susceptible to the impact of environmental factors. The metabolic imprinting during those periods, might influence the future development of obesity from infancy to the onset of adulthood. Epidemiological studies in humans have shown that maternal obesity and diabetes during fetal life and lactation are risk factors for the future development of obesity [22–24]. Likewise, maternal undernutrition was also shown to influence offspring predisposition to metabolic disorders [25, 26].

In the hypothalamus, maternal nutritional insults during development have been shown to affect the function of hypothalamic circuits that regulate energy balance. For instance, maternal undernutrition in rodents alters the activity of appetite and satiety centers in the offspring [27, 28]. This is accompanied by an impairment in neuronal proliferation, axonal elongation and neuropeptide expression in the hypothalamus [29–31]. Similarly, maternal overnutrition has been shown to damage axonal projections in the hypothalamus [32–34]. Hormonal imbalance during pregnancy can also lead to defects in hypothalamic circuits [34, 35]. It is unclear in what way these environmental factors influence the function of brain pathways controlling energy balance. However, over the past two decades, several studies have underscored the importance of epigenetic gene regulation.

## REGULATION OF ENERGY HOMEOSTASIS BY THE BRAIN

The brain acts like the central processing unit of a computer to control energy homeostasis. It integrates internal metabolic signals (i.e. nutrients and hormones) and external sensory cues regarding food availability and palatability [36, 37]. These signals provide information about the type of circulating fuels available in the organism, as well as the amount of energy stored and needed. The areas governing energy balance include important parts of the limbic system, midbrain, brainstem and cortex. However, ≈80 years of research have unquestionably shown that the hypothalamus is the quintessential brain region in the control of homeostatic food intake and energy homeostasis [38].

### Arcuate nucleus of the hypothalamus

The arcuate nucleus (ARC) resides in the medial-basal part of the tuberal hypothalamus, on both sides of the third ventricle (3V) so it receives input from other regions of the brain through the cerebrospinal fluid [39]. The ARC is also in direct contact with the median eminence, an area of the brain where the blood-brain barrier is semi-permeable [40]. This strategic position within the brain, allows the ARC to sense the circulating levels of nutrients and hormones. In the ARC, there are two subsets of neurons playing critical functions in the regulation of appetite and energy expenditure: i) orexigeneic neurons that co-express agouti-related protein (AgRP) and neuropeptide Y (NPY) (hereafter AgRP neurons) and ii) anorexigeneic neurons expressing pro-opimelanocortin (POMC) (hereafter POMC neurons).

### AgRP neurons and the orexigenic pathway

AgRP neurons are stimulated by fasting and send intra-ARC projections to POMC neurons and other hypothalamic structures such as paraventricular nucleus (PVN), lateral hypothalamus (LH) and parabrachial hypothalamus (PBN) [41, 42]. AgRP is an orexigenic (i.e. appetite-stimulating) neuropeptide that is exclusively expressed in the ARC and acts as an inverse agonist to melanocortin receptors MC3R and MC4R [43, 44]. AgRP inhibits α-melanocyte-stimulating hormone (α-MSH) signaling exclusively in the PVN to regulate feeding [45]. Conversely, NPY is widely expressed outside the ARC and exerts its orexigenic effect through NPY receptors (NPY1R to NPY5R) [46]. AgRP and NPY are both primary drivers to initiate food intake, as central injection of either neuropeptide causes hyperphagia [43, 47, 48]. However, deletion of *Agrp* or *Npy* genes in mice have shown no effect on food intake and body weight [49]. Several studies have consolidated our understanding of the function of AgRP neurons. For instance, ablation of AgRP neurons in adult mice results in severe anorexia [42, 50], while acute activation of those neurons by optogenetic or chemogenetic means leads to a robust increase in food intake [51, 52]. In addition, AgRP neurons can inhibit other neurons via γ-aminobutyric acid (GABA) action in the ARC (i.e. POMC neurons) and in other areas of the brain [53–56]. Remarkably, GABAergic inputs from AgRP neurons can modulate food intake by acting in the PVN [53]. These discoveries highlight the importance of GABA signals from AgRP neurons in the regulation of energy balance, through the inhibition of anorexigenic neuronal populations all over the brain [57].

### POMC neurons and the central melanocortin system

POMC neurons project mainly to the PVN but also to the LH, the ventromedial hypothalamus (VMH) and dorsomedial nucleus (DMN) [58]. POMC neurons produce the prohormone POMC, which expression is restricted to the ARC and the nucleus of the solitary tract (NTS) of the brainstem [59, 60]. POMC precursor is cleaved into diverse neuropeptides including α-MSH, which binds MC4Rs resulting in a reduction in appetite and enhanced energy expenditure [44, 61–63]. Consistently, *Pomc* or *Mc4r* deficiency causes hyperphagia and obesity in both mice and humans [16, 17, 64–66]. In addition, β-endorphin (a POMC-derived neuropeptide) is released from ARC neurons and regulates feeding after binding to the opioid receptor [67]. Recent investigations using optogenetic and chemogenetic approaches have confirmed the role of POMC neurons in feeding control and energy homeostasis [52, 68]. Acute chemogenetic stimulation of POMC neurons in the dark phase (a natural feeding period) suppresses food intake whereas consumption of a meal increases their activity, which supports the role for α-MSH in short-term feeding control [69]. However, prolonged activation of POMC neurons is necessary to suppress food intake during the light phase, suggesting that α-MSH might be as well involved in long-term regulation of energy homeostasis [52, 68]. Indeed, deep brain imaging studies have shown that POMC neurons are gradually and persistently depolarized by leptin [37]. Altogether, these data point towards a role of α-MSH in long- and short-term energy balance. At this time, the mechanisms underlying these two distinct effects remain unknown.

POMC, AgRP and MC4R-expressing neurons constitute the central melanocortin system. This is arguably the best-characterized neuronal network involved in energy balance control. The melanocortin system is characteristically composed of fibers that express both agonists (α-MSH) and antagonists (AgRP) of the melanocortin receptors and receives inputs from hormones, nutrients and afferent neural circuits [15, 70–74].

In addition to neuropeptides, hypothalamic neurons can respond to nutrients by modifying the synthesis and/or activity of cellular energy sensors. In the last decades, many evidences have shown that hypothalamic AMP-activated protein kinase (AMPK) is a nutrient and energy sensor that controls whole-body energy homeostasis [75–77]. Nonetheless, in the hypothalamus AMPK integrates the orexigenic and anorexigenic pathways [78]. Genetic evidences have shown that mice lacking *Ampk* in POMC or AgRP neurons display an impaired energy balance alongside alteration on body weight and glucose homestasis [75].

## EPIGENETIC GENE REGULATION

The term *epigenetics* was defined by the developmental biologist Conrad Waddington in 1942 [79]. Currently, the accepted definition of epigenetics is “stably heritable phenotype resulting from changes in a chromosome without alterations in the DNA sequence” [80]. In general, it is acknowledged that epigenetic is an additional regulatory layer for gene expression control. There are several epigenetic modifications that can change chromatin structure, including DNA methylation, post translational modification of histone tails and regulatory RNAs.

### DNA methylation

DNA methylation was the first epigenetic mark discovered [81]. It is a stable covalent modification that mostly occurs on DNA regions where a cytosine is followed by a guanine (CpG) and is catalyzed by a family of enzymes called DNA methyltransferases (DNMT). These enzymes add a methyl group on the 5^th^ carbon of a cytosine to generate a 5-methylcytosine (5-mC). In mammals, there are three DNMTs: DNMT1, DNMT3a and DNMT3b. DNMT1 maintains DNA methylation during replication [82]. DNMT3a and DNMT3b are responsible for *de novo* methylation [83]. DNA methylation has many biological functions including X chromosome inactivation, the monoallelic expression of imprinted genes and transcriptional repression of transposon-derived sequences [84, 85].

For many years, DNA methylation was considered a permanent epigenetic modification that could not be removed. However, this concept has been challenged in recent years with the discovery of ten eleven translocation (TET) enzymes, which catalyze crucial steps for an oxidative demethylation reaction thus providing a mechanistic basis for an active DNA demethylation pathway [86, 87]. In the brain, DNA methylation appears to be particularly important. On one hand, DNMT1 and DNMT3a are highly expressed in post-mitotic neurons when compared to other cell types and double knock-out mice for these proteins show alterations in neuronal plasticity [88]. On the other hand, 5-mC is very abundant in the brain, particularly in the hypothalamus, cortex and hippocampus [89].

DNA methylation is not only restricted to CpG dinucleotides. Several relatively recent studies have revealed high levels of methylation in non-CpG cytosines (mCH, where methylated cytosine is followed by an adenine, thymidine or cytosine) [90–92]. In the brain, methylation of mCH sites is very dynamic when compared to CpG islands and it occurs during central nervous system maturation in the early years of life [90]. These novel insights have raised new and exciting questions regarding the functional role of mCH in the brain.

### Histone post-translational modifications

Chromatin is a complex of DNA wrapped around a nucleosome, which is composed of canonical histones H2A, H2B, H3 and H4 [93]. Nonetheless, DNA has to be accessible to allow molecular processes like transcription, DNA repair and replication. Compelling evidence have shown that post-translational modifications (PTMs) of histone tails can modulate chromatin structure and hence transcriptional activity. Histone tails can undergo a large variety of PTMs including acetylation and methylation among others [94].

Histone acetylation is defined by the addition of an acetyl group on a lysine or an arginine residue of histone tails [95]. It is catalyzed by specific histone acetyltransferases (HAT) [96]. Histone acetylation is reversible, as histone deacetylases (HDACs) can remove the acetyl groups from histone tails. HDACs are classified in four classes (I, II, III and IV) according to their functions and DNA sequence [97]. Histone acetylation plays a role in chromatin assembly and participates in the regulation of gene expression [98]. At the molecular level, it is believed that histone acetylation increases the accessibility of transcription factors to DNA by lowering the affinity between histones and DNA [98].

Histone methylation occurs mainly on arginine, lysine and histidine [99, 100]. Specific histone methyltransferases (HMT) catalyze the addition of one or more methyl groups to histone tails [101]. SET-domain containing, and DOT1-like methyltransferases are specific for lysine, while N-methyltransferases (PRMT) are specific for arginine [102]. Histone methylation was believed to be irreversible until the discovery of the H3K4 lysine-specific demethylase 1A (KDM1A or LSD1) [99, 103]. Since then, many other demethylases have been identified [103, 104]. Overall, histone acetylation and methylation are the most studied histone PTMs, due to their effects in gene expression, and are systematically used to map chromatin structure across the genome.

### Non-coding RNA

Non-coding RNAs (ncRNAs) are relatively new concept in epigenetics. These molecules make up the majority of the transcriptome but, unlike messenger RNA (mRNA), ncRNAs are transcribed from DNA but not translated into protein. There are three different types of ncRNAs [105, 106]: (i) small nuclear RNAs (snRNAs) and small nucleolar RNAs (snoRNAs); (ii) interference RNA, including micro RNAs (miRNAs); and (iii) long ncRNAs (lncRNAs).

snRNAs and snoRNAs are involved in the processing and regulation of other RNAs such as mRNA and ribosomal RNA (rRNA). miRNAs are short ncRNAs (≈22 nucleotides in length) that regulate gene expression via mRNA silencing [105]. Normally, miRNAs bind to complementary mRNA target sequences and either inhibit their translation or cause the degradation of the mRNA [105]. lncRNAs are large RNA molecules localized in the cytoplasm or the nucleus with a length of more than 200 nucleotides. Despite lncRNAs being thought to account for the majority of the ncRNA transcriptome, their discovery is still at a preliminary stage and few lncRNAs have been characterized in detail so far. However, it is clear that lncRNAs are important regulators of gene expression through a wide variety of mechanisms [106, 107].

## EPIGENETICS OF ENERGY BALANCE CONTROL IN THE HYPOTHALAMUS

Numerous studies have revealed that epigenetic mechanisms are involved in many aspects of metabolic dysfunction. Genomic data indicate that obesity and T2D are associated with altered DNA methylation patterns at specific loci [108, 109]. Similarly, growing evidence links PTMs of histone tails with metabolic disease, especially T2D [110]. Moreover, epigenetic modifications can explain the molecular mechanisms underlying fetal programming and its association with metabolic disorders [12, 111].

**Figure 2:**
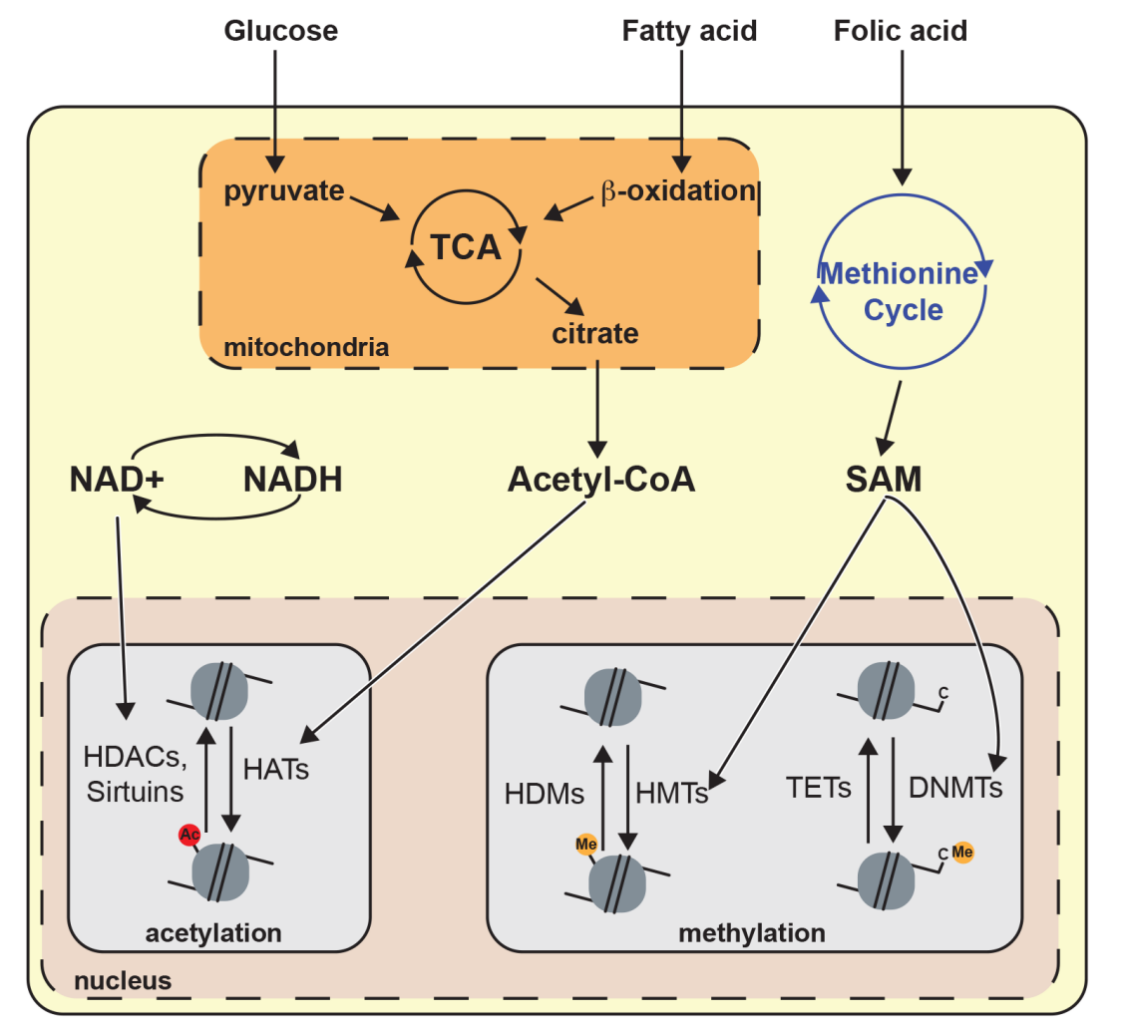
FIGURE 2: Metabolites influence chromatin architecture. Glucose and fatty acid catabolism produce acetyl-CoA through metabolic pathways including tricarboxylic acid cycle (TCA) and β-oxidation. Acetyl-CoA regulates histone acetylation because it is important for the enzymatic activity of histone acetyltransferases. NAD+ is produced by oxidative pathways and is a relevant cofactor for histone deacetylation mediated by sirtuins. The methionine cycle is the principal producer of S-adenosyl methionine (SAM), which is a cofactor for histone/DNA methyltransferase (HMT or DNMT). Histone deacetylase (HDAC), ten-eleven translocation (TET), histone demethylase (HDM).

The epigenetic-dependent regulation of metabolism is reciprocal, as many metabolites and nutrients can serve as substrates/co-factors for epigenetic-modifying enzymes. Therefore, changes in the concentration of particular metabolites should be considered as a novel signaling cue implicated in the control of gene expression (**Figure 2**). For instance, acetyl-CoA derived from glucose and fatty acid metabolism directly impacts the chromatin architecture by modulating the activity of chromatin-modifying enzymes [112, 113]. Moreover, nutrient sensors such as AMPK directly regulate epigenetic processes. For example, activated AMPK can modify the activity of several HATs and therefore impact histone acetylation [114, 115]. Yet, AMPK impact on epigenetic is not limited to histone acetylation as it has been shown to also influence DNA methylation and other histone PTMs [116]. In this context, it seems reasonable that diet insults may cause epigenetic perturbations in the neurons of the brain governing energy balance.

### Hypothalamic DNA methylation and energy balance control

An excellent example of the influence of epigenetic gene regulation is shown by the *Agouti* (*A*^*vy*^) mouse model, in which genetically identical mice can have a completely different phenotype in terms of both color and size. The *Agouti* gene promotes a yellow mouse coat color and affects energy metabolism through inhibition of the melanocortin signaling [117]. This gene is regulated in part by the methylation status of a transposable element (IAP) located in its promoter. Accordingly, mice showing a methylated IAP have a normal body weight and are brown while an unmethylated promoter generates yellow mice that are prone to obesity [118]. In *A*^*vy*^ mice, maternal supplementation with folic acid, a form of vitamin B9 critical for DNA and protein methylation, results in a shift towards the lean phenotype in the offspring [118]. Conversely, fetal or neonatal exposure to the endocrine disruptor bisphenol A is associated with higher body weight and unmethylation of the *Agouti* gene [119].

Maternal undernutrition has been shown to decrease the activity of hypothalamic DNMTs [120]. Maternal stress, which predisposed the female offspring to binge eating (BE)-like behavior, also altered the expression of hypothalamic DNMTs, causing hypomethylation of hypothalamic miR-1a and downstream dysregulation of the melanocortin system [121]. Moreover, these alterations could be reverted by a methyl-balanced diet during puberty [121].

Numerous reports have evaluated the impact of DNA methylation on the expression of key metabolic genes in the hypothalamus. For instance, overfeeding altered the methylation status of *Pomc* promoter in rat [122]. Similarly, maternal undernutrition changed the methylation of *Pomc* promoter in sheep [111, 123]. In addition, *Pomc* promoter methylation was decreased in a model of rats resistant to diet-induced obesity [124]. More importantly, methylation of CpGs at the intron2–exon3 junction of *POMC* gene is higher in obese children as compared to normal-weight individuals. Insulin signaling in the hypothalamus might also be affected by DNA methylation [125]. Plagemann and colleagues reported that methylation in the promoter region of *Insulin receptor* (*InsR*) is higher in the hypothalamus of rats coming from small litters, suggesting that increased glucose levels due to overfeeding in neonates might be the cause [126]. Alterations in the methylation of *Npy* promoter were also observed in the PVN of mice fed on a cafeteria diet [127]. Moreover, genetic studies have shown that deletion of the DNA methyltransferase *Dnmt3a* in PVN Sim1-neurons leads to obesity [128].

Methylated DNA recruits various proteins with a methyl-CpG-binding domain (MBD), such as methyl-CpG-binding protein 2 (MeCP2). A genetic study revealed an important role for MeCP2 in the regulation of energy metabolism, as mice lacking *Mecp2* in Sim1 neurons developed an obese phenotype [129]. Similarly, *Mecp2* deletion in POMC neurons results in increased body weight, fat mass, leptin resistance and food intake [130]. Altogether, these studies have highlighted the crucial role of DNA methylation in the hypothalamic regulation of energy metabolism.

### Hypothalamic histone PTMs and energy balance control

The activity of most chromatin modifiers is influenced by metabolites. Glucose and fatty acid catabolism produce acetyl-CoA, which is an essential acetyl group donor in histone acetylation reactions. Thus, acetyl-CoA links energy metabolism with epigenetic gene regulation [131]. In addition, NAD^+^ is a common molecule in various oxidative pathways and it is also an obligate cofactor for sirtuin-dependent histone deacetylation (**Figure 2**) [132]. Hence, fluctuating NAD^+^ levels could contribute to histone deacetylation by sirtuin. Nonetheless, the connection between histone PTMs in the hypothalamus and obesity predisposition has not been sufficiently explored and remains largely unknown.

Some of the few available studies have focus on the function of histone acetylation in hypothalamic neurons. The first evidence emerged from studies on the (NAD^+^)-dependent class III deacetylase sirtuin 1 (SIRT1). This particular enzyme regulates gene expression by deacetylation of proteins including transcription factors and histones. Importantly, SIRT1 levels are high in the hypothalamus including the ARC and VMH [132]. SIRT1 in the hypothalamus is believed to act as a nutrient sensor, as lack of Sirt1 in SF1 or POMC neurons causes hypersensitivity to high-fat diet and decreased energy expenditure [133, 134]. On the contrary, in orexigenic AgRP neurons SIRT1 deficiency suppresses food intake on a standard diet [135, 136]. Interestingly, *Sirt1* expression was also shown to be affected by aging in the ARC [137], and it has been suggested that conditional knock-in of Sirt1 in AgRP and POMC neurons could protect against aging-associated obesity by inhibiting feeding and stimulating energy expenditure [138]. Nevertheless, the data accumulated so far on the role of SIRT1 in the hypothalamus do not distinguish if its action is mediated through chromatin remodeling or other processes.

Diet insults seem to modulate the hypothalamic expression of several HDACs, such as *Hdac3*, *Hdac4* and *Hdac5* [139]. Indeed, HDAC5 is necessary for correct leptin signaling in hypothalamic neurons. Specifically, HDAC5 regulates the localization of STAT3, a crucial transcription factor that mediates leptin signaling in neurons [140].

### Hypothalamic ncRNAs and energy balance control

**Figure 3:**
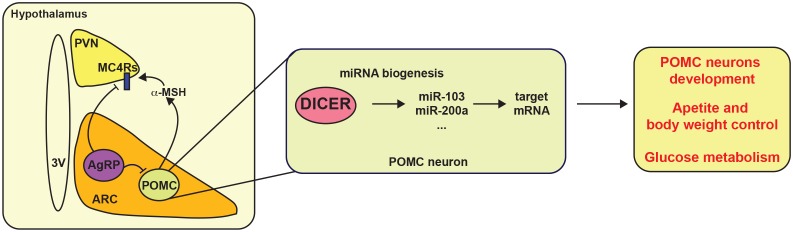
FIGURE 3: Hypothalamic miRNAs control energy balance. POMC and AgRP neurons from the arcuate nucleus (ARC) send projections to the paraventricular nucleus (PVN) to control energy balance. Dicer and several miRNAs, such as miR-103 and miR-200a, have been proposed to control appetite and body weight likely via POMC neurons. Additionally, miRNA biogenesis is also important for the development of POMC neurons. Melanocortin receptor 4 (MC4R).

Many recent studies have suggested that miRNAs might be important regulators of energy balance by modulating the melanocortin system. The first evidence that miRNAs are involved in the hypothalamic control of energy balance came from the observation that expression of *Dicer,* a key gene for miRNAs maturation, is modulated by nutritional status in the hypothalamus [141]. Interestingly, most POMC and AgRP neurons express *Dicer*. Deletion of *Dicer* in POMC neurons causes post-natal neurodegeneration resulting in increased appetite, obesity and T2D (**Figure 3**) [141–143]. In agreement with these observations, brain- and ARC-specific deletion of *Dicer* causes similar metabolic alterations [144, 145]. A recent study has shown that miR-103/107 is potentially involved in the maturation of hypothalamic *Pomc* progenitors [143]. These observations were in accordance with previous studies reporting the importance of miRNAs in neuronal development [146]. In fact, many hypothalamic miRNAs are expressed during development in mice and pig [147, 148]. In the last years, significant effort has been made to identify hypothalamus-specific miRNAs. So far, it has been shown that expression of let-7c, miR-7a, miR-7b, miR-124a, miR-125a, miR-136, miR-138, miR-212, miR-338, miR-451, mir-200a/b and mir-429 is enriched in the hypothalamus [149–152]. Among them, miR-7a displays an interesting pattern of expression that seems to be specific for AgRP and POMC neurons, but its exact function remains elusive [149].

In the hypothalamus, the physiological role of lncRNAs remain elusive and only few studies have focused on them. Brain cytoplasmic RNA 1 (BC1) has been well characterized in neurosecretory axon terminals from the hypothalamus and it regulates protein translation by its binding to the ribosome [153]. Many other lncRNAs have been found to be expressed exclusively in the hypothalamus, yet their function remains completely unknown [154, 155]. One recent study has investigated the relationship between energy availability and lncRNAs in the hypothalamus, revealing that the pattern of expression of many lncRNA is regulated by fasting [156]. Recently, a study in rodents has highlighted the importance of the *Snord116* genomic cluster, a locus encoding multiple ncRNAs, in the hyperphagia observed in Prader-Willi syndrome [157] These data have provided notable information, yet one of the biggest challenges in the field will be to elucidate their precise functions in energy balance control.

## CONCLUSIONS AND FUTURE DIRECTIONS

Obesity is the result of disrupted energy balance, which is partially the consequence of alterations in the hypothalamic melanocortin circuitry. Indeed, various obesity susceptibility genes have been identified and some of them belong to the central melanocortin system. Therefore, a better understanding of the precise mechanisms implicated in the melanocortin control of energy balance is a fundamental requisite for the development of more effective anti-obesity therapeutic strategies. Importantly, genetics alone cannot explain the current obesity epidemics. Overfeeding and the prevailing obesogenic environment can impair the sophisticated hypothalamic circuits that regulate energy homeostasis, and current evidence underscores the importance of epigenetic gene regulation in this process. Indeed, metabolites derived from the diet are necessary for the function of many chromatin modifying enzymes. The reversible nature of most epigenetic modifications makes them very attractive targets for possible anti-obesity intervention and prevention strategies.

The hypothalamus is a complex neuronal network with a remarkable variety of cell populations. It is therefore very likely that AgRP and POMC neurons might have exclusive epigenomes. To date, analysis of epigenetic signatures has been performed in specific gene promoters and whole hypothalamic samples. This represents a major limitation, as it might mask the diversity of cell-specific epigenetic marks. New methodologies such as single-cell technology or laser dissection have been proposed to tackle this limitation. However, the high cost or low yield of these approaches do not overcome this challenge. In moving forward, more studies are necessary to elucidate the role of epigenetics upon appetite and energy balance control in a neuron-specific manner. These answers will be crucial, not only to improve our understanding of the gene-environment interactions, but also for the development of potential epigenetic-based future therapies aimed at controlling food intake and body weight.

## Abbreviations

5-mC: – 5-methylcytosine,
α-MSH: – α-melanocyte-stimulating hormone,
AgRP: – agouti-related protein,
AMPK: – AMP-activated protein kinase,
ARC: – arcuate nucleus,
DNMT: – DNA methyltransferases,
GABA: – γ-aminobutyric acid,
GWAS: – genome-wide association studies,
HAT: – histone acetyltransferase,
HDAC: – histone deacetylase,
LH: – lateral hypothalamus,
lncRNA: – long ncRNA,
MCR: – melanocortin receptor,
miRNA: – micro RNA,
mRNA: – messenger RNA,
ncRNA: – non-coding RNA,
NPY: – neuropeptide Y,
POMC: – pro-opimelanocortin,
PTM: – post-translational modification,
PVN: – paraventricular nucleus,
snoRNA: – small nucleolar RNA,
snRNA: – small nuclear RNA,
T2D: – type 2 diabetes,
VMH: – ventromedial hypothalamus.
